# Effects of milk containing only A2 beta casein versus milk containing both A1 and A2 beta casein proteins on gastrointestinal physiology, symptoms of discomfort, and cognitive behavior of people with self-reported intolerance to traditional cows’ milk

**DOI:** 10.1186/s12937-016-0147-z

**Published:** 2016-04-02

**Authors:** Sun Jianqin, Xu Leiming, Xia Lu, Gregory W. Yelland, Jiayi Ni, Andrew J. Clarke

**Affiliations:** 1 Clinical Nutrition Center, Huadong Hospital affiliated to Fudan University, Shanghai, China; 2 Department of Gastroenterology, Xin Hua Hospital affiliated to Shanghai Jiao Tong University School of Medicine, Shanghai, China; 3 Endoscopic Center, Shanghai International Medicine Center, Shanghai, China; 4 Department of Gastroenterology, Central Clinical School, The Alfred Centre, Monash University, Melbourne, VIC Australia; 5 School of Health Sciences, RMIT University, Bundoora, VIC Australia; 6 S.P.R.I.M. China (Shanghai) Consulting Co., Ltd., Shanghai, China; 7 The a2 Milk Company Limited, Auckland, New Zealand

**Keywords:** β-casein, Cows’ milk, Lactose intolerance, Gastrointestinal function, Cognitive processing

## Abstract

**Background:**

Cows’ milk generally contains two types of β-casein, A1 and A2 types. Digestion of A1 type can yield the peptide β-casomorphin-7, which is implicated in adverse gastrointestinal effects of milk consumption, some of which resemble those in lactose intolerance. This study aimed to compare the effects of milk containing A1 β-casein with those of milk containing only A2 β-casein on inflammation, symptoms of post-dairy digestive discomfort (PD3), and cognitive processing in subjects with self-reported lactose intolerance.

**Methods:**

Forty-five Han Chinese subjects participated in this double-blind, randomized, 2 × 2 crossover trial and consumed milk containing both β-casein types or milk containing only A2 β-casein. Each treatment period was 14 days with a 14-day washout period at baseline and between treatment periods. Outcomes included PD3, gastrointestinal function (measured by smart pill), Subtle Cognitive Impairment Test (SCIT), serum/fecal laboratory biomarkers, and adverse events.

**Results:**

Compared with milk containing only A2 β-casein, the consumption of milk containing both β-casein types was associated with significantly greater PD3 symptoms; higher concentrations of inflammation-related biomarkers and β-casomorphin-7; longer gastrointestinal transit times and lower levels of short-chain fatty acids; and increased response time and error rate on the SCIT. Consumption of milk containing both β-casein types was associated with worsening of PD3 symptoms relative to baseline in lactose tolerant and lactose intolerant subjects. Consumption of milk containing only A2 β-casein did not aggravate PD3 symptoms relative to baseline (i.e., after washout of dairy products) in lactose tolerant and intolerant subjects.

**Conclusions:**

Consumption of milk containing A1 β-casein was associated with increased gastrointestinal inflammation, worsening of PD3 symptoms, delayed transit, and decreased cognitive processing speed and accuracy. Because elimination of A1 β-casein attenuated these effects, some symptoms of lactose intolerance may stem from inflammation it triggers, and can be avoided by consuming milk containing only the A2 type of beta casein.

**Trial registration:**

ClinicalTrials.gov/NCT02406469

**Electronic supplementary material:**

The online version of this article (doi:10.1186/s12937-016-0147-z) contains supplementary material, which is available to authorized users.

## Background

Dairy products, especially those derived from cows’ milk, are a major nutritional component and their consumption continues to increase worldwide. However, the increasing consumption of dairy products is associated with an increase in the risk of or the aggravation of symptoms of some disorders, including gastrointestinal dysfunction [1–5] and immune-/inflammation-related disorders [6, 7]. Some of these effects of dairy products have been attributed to a group of peptides present in milk derived from the proteolysis of β-casein, particularly β-casomorphin-7 (BCM-7).

BCM-7 is uniquely derived from the digestion of the A1 β-casein type but not the A2 β-casein type; the two primary types of β-casein present in milk. Either or both of these types may be expressed in cows’ milk depending on the individual cows’ genetic makeup. Cows may be homozygous for one type, or heterozygous with allelic co-dominance resulting in both types being expressed in milk. The two types differ in their protein structure owing to a substitution of the amino acid at position 67. A2 β-casein and related sub-variants including A3 and D contain a proline residue at this site whereas A1 β-casein and related sub-variants including B and C contain a histidine residue at this position, which allows the preceding seven amino acid residues to be cleaved, yielding BCM-7 [8]. Based on the β-casein structure and potential to yield BCM-7 upon digestion in humans, the β-caseins expressed in human, goat, sheep, and buffalo though not of the A2 type are classed as “A2-like”. It has been reported that casein and its derivatives, particularly BCM-7, exert a variety of effects on gastrointestinal function in animal models, including reducing the frequency and amplitude of intestinal contractions [3, 9–12], increasing mucus secretion [13–15], and suppressing lymphocyte proliferation [16, 17].

Intolerance to dairy products is a commonly reported gastrointestinal disorder, and is usually attributed to lactose intolerance [18]. However, based on the gastrointestinal effects of BCM-7 (and hence milk containing A1 β-casein), it is possible that intolerance to dairy products in some cases is related to the consumption of A1 β-casein rather than lactose per se. Our hypothesis is that the consumption of A1 β-casein leads to the production and exposure of tissue to BCM-7, which exerts a range of pro-inflammatory effects including altered signaling activity, redox disorders, and altered epigenetic regulation of gene expression [19]. A consequence of these changes is the disruption of digestive process, which may manifest as symptoms of lactose intolerance in terms of its presentation. Accordingly, the consumption of milk containing A2 β-casein at the exclusion of A1 β-casein may alleviate or prevent the gastrointestinal disturbances associated with BCM-7.

To date, however, few studies have compared the gastrointestinal effects of milk containing only the A2 β-casein type with milk containing A1 β-casein in humans [20]. Therefore, we performed a randomized, controlled, double-blind crossover study to compare the effects of milk containing only the A2 β-casein type with milk containing the A1 β-casein type in terms of gastrointestinal function, including serum and fecal laboratory tests, gastrointestinal symptoms of post-dairy digestive discomfort, stool frequency, Bristol Stool Scale, gastrointestinal transit time, and gastrointestinal inflammation. We hypothesized that the consumption of milk containing A1 β-casein would lead to systemic inflammation and gastrointestinal disorders similar to those of lactose intolerance in a cohort of subjects with perceived or confirmed lactose intolerance. We also hypothesized that elimination of the A1 β-casein type by providing subjects with milk that only contained the A2 β-casein type would avoid or attenuate these effects of A1 β-casein.

Because milk containing only the A1 β-casein type is not commercially available for consumption and is not representative of consumer milk products, we used normal milk containing a mixture of both the A1 and A2 β-casein types. Milk containing only the A2 β-casein type was confirmed to be prepared from cows homozygous for the A2 genotype.

We focused on a Chinese Han population because of the very high rate of perceived lactose intolerance or reported lactose malabsorption of up to 90 % in this population noted in some studies [21–23]. In spite of this, milk consumption in China has continued to increase, with per capita dairy product consumption among urban residents tripling from nearly 6 kg in 1992 to 18 kg by 2006 [24].

## Methods

### Study design

The study was conducted in Accordance with the Declaration of Helsinki as amended in Seoul 2008 and was approved by the ethics committee of the Shanghai Nutrition Society (approval number: SNSIRB#2014[002]). The study was registered with ClinicalTrials.gov (identifier: NCT02406469). All subjects provided written informed consent prior to inclusion in the study.

**Fig. 1:**
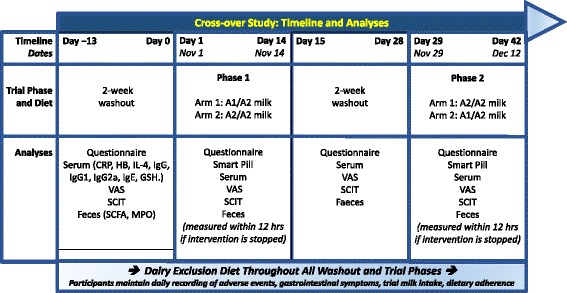
Study design. A1 = milk containing A1 and A2 β-casein; A2 = milk containing only A2 β-casein; hs-CRP, highly sensitive C-reactive protein; Hb, hemoglobin; IL-4, interleukin-4; Ig, immunoglobulin; BCM-7, β-casomorphin-7; GSH, glutathione; PD3, gastrointestinal symptoms of post-dairy digestive discomfort; SCIT, Subtle Cognitive Impairment Test; SCFA, short-chain fatty acids; MPO, myeloperoxidase

This was a single-site, double-blind, randomized, controlled, 2 × 2 cross-over study designed to evaluate the effects of milk containing only the A2 β-casein type versus milk containing the A1 and A2 β-casein types on serum levels of immune response markers in correlation to symptoms of intolerance. The design of the study is shown in Fig. 1. After a screening visit at which the subjects underwent full clinical evaluations and qualitative tests for urinary galactose, eligible subjects entered a 2-week washout period. Then, subjects entered intervention period 1 in which they received milk containing only the A2 β-casein type or milk containing both β-casein types according to the randomization scheme for 2 weeks. After a second 2-week washout period, the subjects entered intervention period 2 in which they received the opposite milk product. Visits were scheduled at the start of each intervention period and at Days 7 and 14 in each intervention period. The subjects were contacted by telephone during each washout period. The study was conducted at the Department of Gastroenterology, Xin Hua Hospital Affiliated to Shanghai Jiao Tong University School of Medicine (Shanghai, China).

### Interventions

Milk containing only the A2 β-casein type and milk containing both the A1 and A2 β-casein types were provided by A2 Infant Nutrition Limited (Auckland, New Zealand), and were distributed to the study site by SPRIM China. Staff at SPRIM China repackaged and labeled all of the products to ensure the investigators and subjects were blinded to which product they received in each intervention period. In each intervention period, the subjects were instructed to consume 250 ml of milk after two meals per day for 14 days. Subjects used a diary to record milk intake and adherence to each intervention. The used and unused cartons were collected at each visit to evaluate compliance to the interventions and to confirm that the blinding was intact.

Subjects were randomized, with stratification by gender, to sequence 1 (A1/A2 → A2) or sequence 2 (A2 → A1/A2) according to the allocation number filed in sealed envelopes. The allocation was based on a computer-generated list prepared by SPRIM China.

The milk containing only the A2 β-casein type contained (per 100 ml) 271 kJ energy, 3.1 g protein, 3.6 g fat, 5.0 g carbohydrate, 48 mg sodium, 150 mg potassium, and 117 mg calcium. The ratio of A1 β-casein to A2 β-casein was approximately 40:60 in milk containing both β-casein types, as confirmed by ultra performance liquid chromatography and mass spectrometry. Both products were identical and contained the same amount of protein.

The consumption of dairy products other than those provided was prohibited during the study and subjects were not permitted to consume any cows’ milk products during each washout period.

### Subjects

The inclusion criteria were as follows: male or female; age 25–68 years; irregular milk consumption (as documented using a food frequency questionnaire); self-reported intolerance to commercial milk; self-reported mild to moderate digestive discomfort after milk consumption; and normal electrocardiograms (ECG) and blood pressure during quiet respiration.

Subjects were enrolled if they: agreed not to take any medication, nutritional supplements, or other dairy products, including acidophilus milk, during the study; were willing to comply with all of the requirements and procedures; provided signed informed consent; agreed not to participate in another interventional clinical research study during the present study; did not meet any of the exclusion criteria (see Additional file 1); and fully understood the nature, objective, benefit, and the potential risks and side effects of the study. No subjects with existing conditions such as irritable bowel syndrome, constipation, or un-medicated inflammatory bowel disease were enrolled.

Subjects were recruited via advertisements placed on noticeboards at community hospitals.

### Study measures

At screening, the subjects underwent comprehensive evaluations, including screening of medical history, measurements (height, body weight, blood pressure, ECG), physical examinations, and quantitative tests for urinary galactose. The following assessments were made at baseline and Day 14 in both intervention periods: the Subtle Cognitive Impairment Test (SCIT); self-reported symptoms of post-dairy digestive discomfort; and laboratory tests (See Additional file 1). Subjects used daily diaries to record milk intake, adherence, gastrointestinal symptoms, and adverse events. In addition, subjects were given a smart pill (OMOM Capsule; Chongqing Jinshan Science & Technology [Group] Co., Ltd., Chongqing, China) on Day 14 of each intervention period.

#### Gastrointestinal symptoms of post-dairy digestive discomfort

Gastrointestinal symptoms were recorded using the Bristol Stool Chart in daily diaries, which included stool frequency and stool consistency. Stool consistency was evaluated on a 7-point scale, ranging from 1 = separate hard lumps that are hard to pass to 7 = watery with no solid pieces or entirely liquid. At each visit, the gastrointestinal symptoms of post-dairy digestive discomfort were assessed by the investigator who asked whether the subject felt any of the following: bloating, abdominal pain, flatulence, heavy stomach and borborygmi (stomach rumbling). Each symptom was rated on a 4-point Likert-type scale, as never (score = 0), rarely (score = 1), frequently (score = 2), or all of the time (score = 3).

#### Measurement of gastrointestinal transit time and inflammation using a smart pill

The smart pill was given on Day 14 of each intervention period to calculate the following variables: small bowel transit time (SBTT)—the time between capsule entry into the small bowel and entry into the cecum; colonic transit time (CTT)—time between the entry into the cecum and defecation; and whole gastrointestinal transit time (WGTT)—time between capsule ingestion and defecation.

Stomach and small bowel inflammation was also evaluated using the smart pill, and graded as improved, worsened, and unchanged. As a pilot trial, a limited number of smart pills were purchased. Inflammation was diagnosed and assessed by a gastroenterologist using images obtained using the smart pill. Inflammation was graded as worse/not at all/better based on the images and videos obtained after the two interventions. The gastroenterologist was blinded to the intervention and was only provided with the subjects’ identification numbers.

#### SCIT

The SCIT is a computer-based test that measures the speed and effectiveness of information processing [25]. Participants indicate which of the two parallel vertical lines in the target stimulus is shorter by pressing the left or right mouse button. A visually masked target stimulus is randomly presented at exposure durations of 16, 32, 48, 64, 80, 96, 112 and 128 ms; 12 trials at each for a total of 96 trials. Subject response time and error rate are recorded for stimulus exposure duration. Data for the four shortest exposure durations 16–64 ms; referred to as the “head” of the response curve) are pooled to provide two representative test scores for pre-conscious-automatic processing: response time (SCIT-RTH) and error rate (SCIT-EH). Data for the four longer presentation durations (83–133 ms; referred to as the “tail” of the response curve) are pooled to provide two more representative scores for conscious processing: response time (SCIT-RTT) and error rate (SCIT-ET). The SCIT has high test–retest and internal consistency reliabilities, and medium–high content validity [25].

#### Adverse events

Adverse events were recorded using case report forms and categorized in terms of their severity, potential relationship to the interventions, and outcome. The type of event was recorded using the codes presented in Additional file 1.

### Statistical analysis

As an exploratory study, sample size calculations were not performed. We planned to recruit approximately 40 subjects after considering the design and results of a prior double-blind, randomized, 8-week cross-over trial [20].

The Kolmogorov–Smirnov Test was used to assess the normality of continuous variables. Non-normally distributed variables were subjected to square-root or log transformation to approximate a normal distribution. Baseline characteristics are presented descriptively as means ± standard deviation (SD) or the number (percent) of subjects. SCIT, gastrointestinal transit times, stool frequency/consistency, and laboratory variables were analyzed using mixed-effects analysis of variance in which the allocated intervention and intervention period were included as fixed effects, and subject was included as a random effect nested within the study sequence (i.e., sequence 1, A1/A2 → A2; sequence 2, A2 → A1/A2). To investigate whether there were differences between the two interventions in the mean values for each endpoint, and whether the mean values changed during the study periods, Type III tests of fixed effects were used to tests the effects of the interventions and study periods. Additionally, contrast tests were performed to compare the mean values for each product. The presence of a carry-over effect was evaluated using the interaction Intervention × Period. If this interaction was not significant, data from both periods were evaluated. If the interaction was significant, only data from intervention period 1 were used. Gastrointestinal symptoms and results of the urinary galactose test were evaluated using generalized estimating equations (GEEs) in which intervention sequence and measurement time were included as fixed effects and subject was included as a random effect nested within the study sequence. No adjustments were made for multiple comparisons. Adverse events are reported in terms of the number (percent) of subjects with each type of event.

## Results

### Subjects

This study was performed between October 2014 and December 2014. Overall, 104 subjects agreed to participate in the study and underwent the urinary galactose test. All of the subjects were Chinese. Of these, 45 subjects (21 males, 24 females; mean ± SD age 46.6 ± 14.0 years) with self-reported intolerance to cows’ milk satisfied the eligibility criteria and were randomized to sequences 1 or 2. Twenty-three subjects (8 in sequence 1 and 15 in sequence 2) were confirmed to be lactose intolerant based on the results of the urinary galactose test. The subjects allocated to both sequences were well matched in terms of their baseline characteristics (Table 1). All of the subjects reported that they did not regularly consume cows’ milk and had self-reported intolerance to cows’ milk.

**Table 1:** Subject characteristics

Study group		Sequence 1 (*n* = 22)^a^		Sequence 2 (*n* = 23)^b^		All subjects		*P*-value^c^
Gender	Male	10	(45.5 %)	11	(47.8 %)	21	(46.7 %)	—
	Female	12	(54.5 %)	12	(52.2 %)	24	(53.3 %)	
Age (year)		45.7	(12.3)	47.5	(15.6)	46.6	(14.0)	0.664
Weight (kg)		72.4	(19.9)	66.7	(14.3)	69.5	(17.3)	0.272
Height (cm)		167.5	(9.4)	166.4	(8.0)	166.9	(8.6)	0.695
BMI (kg/m^2^)		25.4	(4.6)	24.0	3.7	24.6	4.2	0.226
Body temperature (°C)		36.9	(0.1)	36.8	(0.2)	36.8	(0.2)	0.207
DBP (mmHg)		76.1	(5.2)	75.5	(6.5)	75.8	(5.8)	0.748
SBP (mmHg)		124.6	(6.7)	121.2	(8.8)	122.9	(7.9)	0.145
Lactose intolerant		8	(36.4 %)	15	(65.2 %)	23	(51.1 %)	

^a^Sequence 1: A1/A2 → A2

^b^Sequence 2: A2 → A1/A2

^c^ANOVA

*BMI* body mass index; *DBP* diastolic blood pressure; *SBP* systolic blood pressure

### Serum and fecal biomarkers

Results of the serum and fecal laboratory tests are presented in Table 2. There were no intervention period or sequence effects for any of the laboratory variables (data not shown). However, the baseline value was a significant covariate for all laboratory variables. As shown in Table 2, there were significant differences between the two milk products in terms of the serum concentrations of IL-4 (*P* < 0.0001), IgG (*P* = 0.0007), IgE (*P* = 0.0253), and IgG1 (*P* = 0.0037) and the fecal concentrations of acetic acid (*P* = 0.0052), butanoic acid (*P* = 0.0001), and total short-chain fatty acids (SCFAs) (*P* = 0.0009).

**Table 2:** Results of serum and fecal laboratory tests

Variable	Sequence 1^a^				Sequence 2^b^				Mixed-effects ANOVA		
	Period 1		Period 2		Period 1		Period 2		Estimate^c^	SD	*P*-value^d^
	BL	PI	BL	PI	BL	PI	BL	PI			
Serum											
hs-CRP (mg/L)	1.00 ± 0.70	1.17 ± 0.64	0.97 ± 0.58	1.10 ± 0.58	1.03 ± 1.03	1.02 ± 1.11	1.01 ± 0.98	1.18 ± 1.04	0.0722^e^	0.03746	0.0608
Hb (g/L)	141.7 ± 17.5	145.1 ± 17.0	136.7 ± 23.2	143.9 ± 16.4	142.8 ± 20.1	145.5 ± 17.7	137.5 ± 25.2	142.0 ± 18.1	−0.8654	1.6781	0.6088
IL-4 (ng/L)	11.8 ± 4.2	14.1 ± 5.2	11.1 ± 3.4	11.0 ± 3.2	11.9 ± 4.3	12.0 ± 3.7	11.8 ± 3.4	14.1 ± 4.6	2.5258	0.5338	**<0.0001**
IgG (g/L)	10.3 ± 2.1	11.6 ± 2.3	10.2 ± 1.7	10.6 ± 1.4	10.6 ± 2.1	11.1 ± 1.9	10.8 ± 1.8	12.2 ± 1.7	0.1426^e^	0.03915	**0.0007**
IgE (IU/mL)	61.3 ± 29.0	69.8 ± 38.0	63.3 ± 30.1	66.2 ± 28.9	58.6 ± 31.2	60.7 ± 33.3	56.7 ± 31.3	64.4 ± 34.2	5.9688	2.5741	**0.0253**
IgG1 (μg/mL)	29.4 ± 31.3	37.4 ± 39.1	31.0 ± 33.1	30.3 ± 32.9	33.0 ± 28.3	28.5 ± 28.5	32.9 ± 27.2	37.4 ± 31.4	0.2424^f^	0.07873	**0.0037**
Feces											
Acetic acid (%)	0.42 ± 0.15	0.42 ± 0.15	0.40 ± 0.14	0.46 ± 0.11	0.39 ± 0.19	0.46 ± 0.19	0.39 ± 0.17	0.36 ± 0.11	−0.0667	0.0226	**0.0052**
Propanoic acid (%)	0.18 ± 0.07	0.18 ± 0.07	0.17 ± 0.07	0.17 ± 0.07	0.17 ± 0.09	0.19 ± 0.13	0.18 ± 0.09	0.17 ± 0.07	−0.006^e^	0.0187	0.7504
Butanoic acid (%)	0.17 ± 0.07	0.16 ± 0.07	0.16 ± 0.07	0.20 ± 0.08	0.17 ± 0.09	0.23 ± 0.09	0.17 ± 0.08	0.16 ± 0.05	−0.0515	0.0122	**0.0001**
Total SCFA (%)	0.76 ± 0.24	0.76 ± 0.24	0.72 ± 0.24	0.83 ± 0.19	0.73 ± 0.33	0.88 ± 0.33	0.74 ± 0.28	0.69 ± 0.18	−0.1289	0.03609	**0.0009**

^a^Sequence 1: A1/A2 → A2

^b^Sequence 2: A2 → A1/A2

^c^A1/A2 − A2

^d^Values in bold are statistically significant at *P* < 0.05

^e^Because the variable was non-normally distributed, mixed-effects ANOVA was performed using the square root-transformed values

^f^Because the variable was non-normally distributed, mixed-effects ANOVA was performed using the log-transformed values

*ANOVA* analysis of variance, *BL* baseline, *PI* postintervention (i.e., after 2 weeks of each intervention), *SD* standard deviation, *hs-CRP* highly sensitive C-reactive protein, *Hb* hemoglobin, *BCM-7* β-casomorphin-7, *IL-4* interleukin-4, *Ig* immunoglobulin, *SCFA* short-chain fatty acids

### Gastrointestinal symptoms of post-dairy digestive discomfort

The self-reported gastrointestinal symptoms are summarized in Tables 3 and 4, while the Bristol Stool Scale scores are presented in Table 5. GEE analyses revealed no significant sequence effects on any of the symptoms. There were significant differences in the distributions of the symptom scores for bloating, flatulence and borborygmus in both sequences when the subjects consumed milk containing both β-casein types at W1 and W2 for sequence 1 or W5 and W6 for sequence 2 compared with baseline. The results indicate that the symptoms were worse at these times than at baseline. By contrast, there was no apparent worsening in symptoms when the subjects consumed the milk containing only the A2 type, indicating this type did not influence gastrointestinal symptoms. For stool frequency, Time (*P* < 0.0001) and Sequence × Time (*P* < 0.0001) were significant factors in the mixed-effects ANOVA, but Sequence (*P* = 0.2801) was not. For the Bristol Stool Consistency score, only the interaction Sequence × Time (*P* = 0.0022) was a significant factor. The consumption of milk containing both β-casein types was also associated with increases in both stool frequency and Bristol Stool Scale scores compared with baseline (Tables 2 and 4). By contrast, consumption of milk containing only the A2 β-casein type was not associated with changes in either variable over time.

**Table 3:** Gastrointestinal symptoms, weekly stool frequency, and Bristol Stool Scale scores

Sequence^a,b^	Level	Baseline	Phase 1		Washout		Phase 2	
		Week 0	Week 1	Week 2	Week 3	Week 4	Week 5	Week 6
Bloating								
Sequence 1	0	19 (86.4)	12 (54.6)	12 (54.6)	19 (86.4)	20 (90.9)	19 (86.4)	21 (95.5)
	1	2 (9.1)	6 (27.3)	7 (31.8)	3 (13.6)	2 (9.1)	3 (13.6)	1 (4.6)
	2	1 (4.6)	3 (13.6)	3 (13.6)	0 (0.0)	0 (0.0)	0 (0.0)	0 (0.0)
	3	0 (0.0)	1 (4.6)	0 (0.0)	0 (0.0)	0 (0.0)	0 (0.0)	0 (0.0)
Sequence 2	0	20 (87.0)	19 (82.6)	19 (82.6)	20 (87.0)	20 (87.0)	12 (52.2)	12 (52.2)
	1	2 (8.7)	3 (13.0)	4 (17.4)	3 (13.0)	3 (13.0)	6 (26.1)	9 (39.1)
	2	1 (4.4)	1 (4.4)	0 (0.0)	0 (0.0)	0 (0.0)	4 (17.4)	1 (4.4)
	3	0 (0.0)	0 (0.0)	0 (0.0)	0 (0.0)	0 (0.0)	1 (4.4)	1 (4.4)
Abdominal pain								
Sequence 1	0	19 (86.4)	17 (77.3)	15 (68.2)	18 (81.8)	20 (90.9)	19 (86.4)	20 (90.9)
	1	3 (13.6)	5 (22.7)	7 (31.8)	4 (18.2)	2 (9.1)	3 (13.6)	2 (9.1)
Sequence 2	0	21 (91.3)	22 (95.7)	21 (91.3)	21 (91.3)	21 (91.3)	17 (73.9)	18 (78.3)
	1	2 (8.7)	1 (4.4)	2 (8.7)	2 (8.7)	2 (8.7)	5 (21.7)	4 (17.4)
	2	0 (0.0)	0 (0.0)	0 (0.0)	0 (0.0)	0 (0.0)	1 (4.4)	1 (4.4)
Flatulence								
Sequence 1	0	19 (86.4)	15 (68.2)	15 (68.2)	20 (90.9)	21 (95.5)	21 (95.5)	21 (95.5)
	1	3 (13.6)	4 (18.2)	2 (9.1)	1 (4.6)	0 (0.0)	0 (0.0)	0 (0.0)
	2	0 (0.0)	3 (13.6)	5 (22.7)	1 (4.6)	1 (4.6)	1 (4.6)	1 (4.6)
Sequence 2	0	20 (87.0)	20 (87.0)	20 (87.0)	21 (91.3)	20 (87.0)	3 (13.0)	16 (69.6)
	1	2 (8.7)	2 (8.7)	3 (13.0)	2 (8.7)	3 (13.0)	8 (34.8)	3 (13.0)
	2	1 (4.4)	1 (4.4)	0 (0.0)	0 (0.0)	0 (0.0)	2 (8.7)	4 (17.4)
Heavy stomach								
Sequence 1	0	1 (4.6)	1 (4.6)	2 (9.1)	2 (9.1)	2 (9.1)	6 (27.3)	6 (27.3)
	1	13 (59.1)	15 (68.2)	12 (54.6)	12 (54.6)	13 (59.1)	5 (22.7)	5 (22.7)
	2	8 (36.4)	6 (27.3)	8 (36.4)	8 (36.4)	7 (31.8)	11 (50.0)	11 (50.0)
Sequence 2	0	2 (8.7)	1 (4.4)	3 (13.0)	2 (8.7)	2 (8.7)	3 (13.0)	3 (13.0)
	1	13 (56.5)	15 (65.2)	13 (56.5)	14 (60.9)	14 (60.9)	13 (56.5)	12 (52.2)
	2	8 (34.8)	6 (26.1)	6 (26.1)	7 (30.4)	7 (30.4)	7 (30.4)	8 (34.8)
	3	0 (0.0)	1 (4.4)	1 (4.4)	0 (0.0)	0 (0.0)	0 (0.0)	0 (0.0)
Borborygmus								
Sequence 1	0	15 (68.2)	8 (36.4)	10 (45.5)	15 (68.2)	14 (63.6)	15 (68.2)	13 (59.1)
	1	6 (27.3)	10 (45.5)	5 (22.7)	6 (27.3)	7 (31.8)	7 (31.8)	9 (40.96)
	2	1 (4.6)	4 (18.2)	6 (27.3)	1 (4.6)	1 (4.6)	0 (0.0)	0 (0.0)
	3	0 (0.0)	0 (0.0)	1 (4.6)	0 (0.0)	0 (0.0)	0 (0.0)	0 (0.0)
Sequence 2	0	16 (69.6)	15 (65.2)	16 (69.6)	14 (60.9)	16 (69.6)	6 (26.1)	8 (34.8)
	1	6 (26.1)	8 (34.8)	7 (30.4)	9 (39.1)	7 (30.4)	11 (47.8)	9 (39.1)
	2	1 (4.4)	0 (0.0)	0 (0.0)	0 (0.0)	0 (0.0)	6 (26.1)	6 (26.1)
Weekly stool frequency								
Sequence 1 (mean ± SD)		7.86 ± 1.98	10.95 ± 3.54	11.05 ± 4.21	9.41 ± 2.46	7.95 ± 2.30	8.32 ± 1.70	7.91 ± 1.15
Sequence 2 (mean ± SD)		7.57 ± 1.95	7.91 ± 1.28	7.87 ± 1.91	7.61 ± 1.73	7.83 ± 1.59	10.22 ± 4.16	10.43 ± 3.46
Bristol Stool Consistency score								
Sequence 1 (mean ± SD)		4.05 ± 0.65	4.54 ± 0.77	4.42 ± 0.74	4.28 ± 0.45	4.08 ± 0.46	4.07 ± 0.35	4.05 ± 0.25
Sequence 2 (mean ± SD)		4.09 ± 0.67	4.12 ± 0.33	4.08 ± 0.61	4.12 ± 0.54	4.07 ± 0.51	4.49 ± 0.70	4.35 ± 1.11

Values are presented as the *n* (%) of subjects

^a^Sequence 1: A1/A2 → A2

^b^Sequence 2: A2 → A1/A2

**Table 4:** Effects of sequence, time, and intervention on gastrointestinal symptoms

Outcome	Effect^a,b^		Estimate (log odds ratio)^c^	SE	95 % confidence limit		*P*-value^d^
					Lower	Upper	
Bloating	Sequence 1 vs. 2		0.0522	0.8788	−1.6701	1.7745	0.9526
	Sequence 1	W1 vs. BL	1.7527	0.6363	0.5055	2.9999	**0.0059**
		W2 vs. BL	1.6511	0.6742	0.3297	2.9724	**0.0143**
		W3 vs. BL	−0.051	0.5686	−1.1655	1.0635	0.9285
		W4 vs. BL	−0.496	0.8014	−2.0667	1.0748	0.536
		W5 vs. BL	−0.051	0.7783	−1.5765	1.4745	0.9477
		W6 vs. BL	−1.2279	1.2429	−3.6639	1.2082	0.3232
	Sequence 2	W1 vs. BL	0.3289	0.5601	−0.7689	1.4267	0.5571
		W2 vs. BL	0.2792	0.5768	−0.8513	1.4097	0.6283
		W3 vs. BL	−0.0479	0.5305	−1.0876	0.9918	0.928
		W4 vs. BL	−0.0479	0.5305	−1.0876	0.9918	0.928
		W5 vs. BL	1.9572	0.6713	0.6415	3.2729	**0.0036**
		W6 vs. BL	1.7308	0.6743	0.4092	3.0525	0.0103
Abdominal pain	Sequence 1 vs. 2		0.5028	0.9654	−1.3893	2.3949	0.6025
	Sequence 1	W1 vs. BL	0.6221	0.6176	−0.5884	1.8325	0.3138
		W2 vs. BL	1.0837	0.5145	0.0754	2.092	0.0352
		W3 vs. BL	0.3417	0.5903	−0.8152	1.4987	0.5626
		W4 vs. BL	−0.4568	0.4501	−1.339	0.4255	0.3103
		W5 vs. BL	0	0.5458	−1.0698	1.0698	1
		W6 vs. BL	−0.4568	0.4501	−1.339	0.4255	0.3103
	Sequence 2	W1 vs. BL	−0.735	1.3002	−3.2834	1.8134	0.5719
		W2 vs. BL	0	1.0937	−2.1436	2.1436	1
		W3 vs. BL	0	1.0937	−2.1436	2.1436	1
		W4 vs. BL	0	1.0937	−2.1436	2.1436	1
		W5 vs. BL	1.3386	0.9439	−0.5114	3.1886	0.1562
		W6 vs. BL	1.1058	0.8062	−0.4742	2.6859	0.1702
Flatulence	Sequence 1 vs. 2		0	0.8711	−1.7074	1.7074	1
	Sequence 1	W1 vs. BL	1.0987	0.471	0.1756	2.0218	**0.0197**
		W2 vs. BL	1.2275	0.461	0.324	2.131	**0.0078**
		W3 vs. BL	−0.3793	0.8025	−1.9521	1.1936	0.6365
		W4 vs. BL	−1.0965	1.2459	−3.5384	1.3454	0.3788
		W5 vs. BL	−1.0965	1.2459	−3.5384	1.3454	0.3788
		W6 vs. BL	−1.0965	1.2459	−3.5384	1.3454	0.3788
	Sequence 2	W1 vs. BL	0	0.5227	−1.0244	1.0244	1
		W2 vs. BL	−0.0494	0.5296	−1.0874	0.9885	0.9256
		W3 vs. BL	−0.4911	0.4293	−1.3325	0.3502	0.2526
		W4 vs. BL	−0.0494	0.5296	−1.0874	0.9885	0.9256
		W5 vs. BL	1.5444	0.5457	0.4748	2.6141	**0.0047**
		W6 vs. BL	1.1939	0.541	0.1336	2.2542	0.0273
Heavy stomach	Sequence 1 vs. 2		0.1422	0.5364	−0.9091	1.1935	0.791
	Sequence 1	W1 vs. BL	−0.2612	0.2897	−0.829	0.3067	0.3673
		W2 vs. BL	−0.0935	0.3705	−0.8197	0.6326	0.8007
		W3 vs. BL	−0.0935	0.308	−0.6972	0.5102	0.7614
		W4 vs. BL	−0.2295	0.3383	−0.8925	0.4334	0.4974
		W5 vs. BL	−0.0587	0.3931	−0.8292	0.7117	0.8812
		W6 vs. BL	−0.0587	0.3928	−0.8286	0.7111	0.8811
	Sequence 2	W1 vs. BL	0.0167	0.3576	−0.6841	0.7176	0.9627
		W2 vs. BL	−0.215	0.3906	−0.9806	0.5507	0.5821
		W3 vs. BL	−0.1475	0.3687	−0.8702	0.5752	0.6891
		W4 vs. BL	−0.1475	0.3717	−0.8761	0.5811	0.6915
		W5 vs. BL	−0.2664	0.5159	−1.2776	0.7448	0.6056
		W6 vs. BL	−0.1131	0.5352	−1.1621	0.9358	0.8326
Borborygmus	Sequence 1 vs. 2		0.0635	0.6389	−1.1887	1.3157	0.9208
	Sequence 1	W1 vs. BL	1.337	0.4883	0.3799	2.2941	**0.0062**
		W2 vs. BL	1.4152	0.5307	0.3751	2.4553	**0.0077**
		W3 vs. BL	0	0.4311	−0.845	0.845	1
		W4 vs. BL	0.1847	0.456	−0.7092	1.0785	0.6856
		W5 vs. BL	−0.0619	0.4887	−1.0198	0.896	0.8993
		W6 vs. BL	0.2874	0.5223	−0.7362	1.311	0.5821
	Sequence 2	W1 vs. BL	0.1243	0.3335	−0.5293	0.7779	0.7094
		W2 vs. BL	−0.0606	0.2848	−0.6189	0.4977	0.8315
		W3 vs. BL	0.2951	0.2606	−0.2157	0.8058	0.2575
		W4 vs. BL	−0.0606	0.399	−0.8426	0.7214	0.8793
		W5 vs. BL	2.064	0.4595	1.1634	2.9647	**<0.0001**
		W6 vs. BL	1.7846	0.4922	0.8199	2.7494	**0.0003**

^a^Sequence 1: A1/A2 → A2

^b^Sequence 2: A2 → A1/A2

^c^Baseline − specified visit

^d^Values in bold are statistically significant at *P* < 0.05

*SE* standard error, *BL* baseline, *W* week

**Table 5:** Mixed-effects ANOVA of stool frequency and Bristol Stool Scale scores

Outcome	Sequence^a,b^	Contrast	Estimate^c^	*P*-value^d^	*P*-value^e^
Stool frequency	Sequence 1	W1 vs. BL	3.09 ± 3.05	**<0.0001**	
		W2 vs. BL	3.18 ± 3.79	**<0.0001**	
		W3 vs. BL	1.55 ± 2.32	**0.0127**	
		W4 vs. BL	0.09 ± 2.11	0.8828	
		W5 vs. BL	0.45 ± 1.97	0.4613	
		W6 vs. BL	0.05 ± 1.96	0.9412	
	Sequence 2	W1 vs. BL	0.35 ± 1.53	0.5642	**<0.001**
		W2 vs. BL	0.30 ± 2.06	0.6139	**0.003**
		W3 vs. BL	0.04 ± 2.10	0.9425	**0.028**
		W4 vs. BL	0.26 ± 1.54	0.6654	0.759
		W5 vs. BL	2.65 ± 3.83	**<0.0001**	**0.021**
		W6 vs. BL	2.87 ± 3.21	**<0.0001**	**0.001**
Bristol Stool Consistency score	Sequence 1	W1 vs. BL	0.49 ± 0.86	**0.0031**	
		W2 vs. BL	0.37 ± 0.91	**0.0261**	
		W3 vs. BL	0.23 ± 0.65	0.1588	
		W4 vs. BL	0.03 ± 0.80	0.8445	
		W5 vs. BL	0.03 ± 0.62	0.8753	
		W6 vs. BL	0.01 ± 0.61	0.9687	
	Sequence 2	W1 vs. BL	0.04 ± 0.57	0.818	**0.041**
		W2 vs. BL	−L vs ± 0.67	0.9694	0.120
		W3 vs. BL	0.04 ± 0.72	0.818	0.344
		W4 vs. BL	−L vs ± 0.73	0.9084	0.826
		W5 vs. BL	0.40 ± 0.85	**0.0132**	0.097
		W6 vs. BL	0.26 ± 1.14	0.108	0.358

^a^Sequence 1: A1/A2 → A2

^b^Sequence 2: A2 → A1/A2

^c^Specified visit – baseline (mean ± standard deviation)

^d^Versus baseline; values in bold are statistically significant at *P* < 0.05

^e^Versus sequence 1; values in bold are statistically significant at *P* < 0.05

*SD* standard deviation, *W* week, *BL* baseline

### Gastrointestinal transit times

**Fig. 2:**
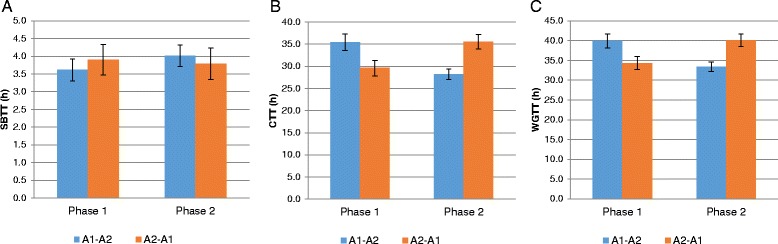
Regional gastrointestinal transit time measured using the smart pill. Values are means ± standard deviation. A1 = milk containing A1 and A2 β-casein; A2 = milk containing only A2 β-casein; CTT = colon transit time; SBTT = small bowel transit time; WGTT = whole gastrointestinal transit time

Because only 80 smart pills were purchased for this study, a smart pill was not given to five subjects in sequence 2 (A2 → A1/A2). Figure 2 compares the regional gastrointestinal transit times measured using the smart pill. Type III tests of fixed effects confirmed that the intervention was a significant factor in terms of CTT (*P* < 0.0001) and WGTT (*P* < 0.0001) but not SBTT (*P* = 0.5930). Intervention period and sequence were not significant factors, indicating that these factors did not influence gastrointestinal transit time. Consumption of milk containing both β-casein types was associated with significantly longer CTT (by 6.6 h, *P* < 0.0001) and WGTT (by 6.3 h, *P* < 0.0001), but not SBTT (−0.20 h, *P* = 0.5903) compared with milk containing only the A2 β-casein type.

### Gastrointestinal inflammation

Smart pill data for gastrointestinal inflammation were available for 22 subjects in sequence 1 (A1/A2 → A2) and 18 subjects in sequence 2 (A2 → A1/A2). Between phases 1 and 2, small bowel inflammation was rated as improved, unchanged, and worsened in 8 (36.4 %), 14 (63.6 %), and 0 (0 %) subjects, respectively, in sequence 1 (A1/A2 → A2) compared with 2 (11.1 %), 15 (83.3 %), and 1 (5.6 %) subjects, respectively, in sequence 2 (A2 → A1/A2) (*P* = 0.042). Between phases 1 and 2, stomach inflammation was rated as improved, unchanged and worsened in 5 (22.7 %), 17 (77.3 %), and 0 (0 %) subjects, respectively, in sequence 1 compared with 2 (11.1 %), 16 (83.3 %), and 0 (0 %) subjects, respectively, in sequence 2 (*P* = 0.427). These results indicate that small bowel inflammation improved in 36.4 % of subjects and stomach inflammation improved in 22.7 % of subjects after switching from milk containing A1/A2 β-casein to milk containing only A2 β-casein. By contrast, small bowel inflammation and stomach inflammation improved in 11.1 % of subjects after switching from milk containing A2 β-casein to milk containing A1 and A2 β-casein.

### SCIT

**Fig. 3:**
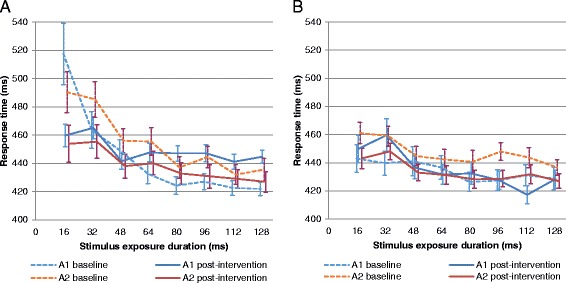
SCIT response times according to the intervention received in phase 1 (**a**); phase 2 (**b**). A1 = milk containing A1 and A2 β-casein; A2 = milk containing only A2 β-casein

Results of the SCIT are presented in Fig. 3a for phase 1 and Fig. 3b for phase 2 for each sequence. Mixed-effects ANOVA confirmed that the intervention was a significant factor in terms of the response time (*P* = 0.0013) and error rate (*P* = 0.0004) for each exposure duration. Significant intervention effects were also found in the tail mean response time (*P* = 0.027) and in the head mean error rate (*P* = 0.020) (Table 6). The baseline values for all SCIT variables were significant covariates, but no statistically meaningful differences were observed between intervention periods or sequence.

**Table 6:** Results of mixed-effects ANOVA for SCIT variables

Variable	Estimate^a^	SD	*P*-value^b^
Response time	8.5798	2.6605	**0.0013**
Head mean response time	10.8330	9.0995	0.2405
Tail mean response time	14.7353	6.4309	**0.0270**
Error rate	1.759 %	0.496 %	**0.0004**
Head mean error rate	2.758 %	1.140 %	**0.0200**
Tail mean error rate	0.402 %	0.529 %	0.4514

^a^Least squares mean difference (A1/A2 − A2)

^b^Values in bold are statistically significant at *P* < 0.05

SD, standard deviation

### Subgroup analysis in subjects with confirmed lactose intolerance

**Fig. 4:**
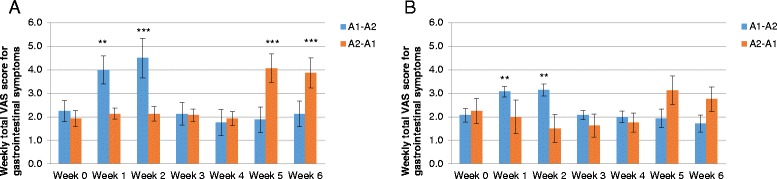
Weekly total gastrointestinal symptom scores (overall) in subjects with (**a**) or without (**b**) lactose intolerance. A1 = milk containing A1 and A2 β-casein; A2 = milk containing only A2 β-casein. **P < 0.01 vs. baseline; **P < 0.001 vs. baseline

Twenty-three subjects were confirmed to be lactose intolerant based on urinary galactose tests. The baseline characteristics of these subjects were similar to those of the lactose tolerant subjects. The weekly total scores for gastrointestinal symptoms of post-dairy digestive discomfort in subjects with or without lactose intolerance are presented in Fig. 4. Consumption of milk containing both β-casein types was associated with significant worsening of gastrointestinal symptoms in lactose intolerant and lactose tolerant individuals in either sequence. By contrast, consumption of milk containing only A2 β-casein was not associated with worsening of gastrointestinal symptoms, as the symptoms were comparable to those observed after the baseline washout of dairy products. When we pooled data from both sequences, the magnitude of the increase in gastrointestinal symptom scores following the consumption of milk containing both β-casein types tended to be greater in lactose intolerant subjects than in lactose tolerant subjects, and this was of borderline significance (least squares mean difference: 1.087; 95 % CI −0.0652, 2.2392; *P* = 0.0638). By contrast, the gastrointestinal symptom score was not significantly different between lactose intolerant subjects and lactose tolerant subjects after the consumption of milk containing only the A2 β-casein type (least squares mean difference: 0.494; 95 % CI −0.3247, 1.3128; *P* = 0.2303). The consumption of milk containing both β-casein types was associated with significant increases in CTT and WGTT, but not SBTT, as compared with milk containing only the A2 β-casein type (Table 7).

**Table 7:** Gastrointestinal transit time in subjects with or without lactose intolerance

	Variable	Sequence 1 (*n* = 22)^a^		Sequence 2 (*n* = 18)^b^		Estimate^c^	SD	*P*-value^d^
		Phase 1	Phase 2	Phase 1	Phase 2			
Overall (*n* = 40)	SBTT (h)	3.62 ± 1.46	4.02 ± 1.45	3.90 ± 1.85	3.79 ± 1.89	−0.1997	0.3704	0.593
	CTT (h)	35.41 ± 8.68	28.23 ± 5.50	29.62 ± 7.41	35.51 ± 6.92	6.6173	1.2916	**<0.0001**
	WGTT (h)	39.95 ± 8.45	33.41 ± 5.68	34.36 ± 6.90	40.14 ± 6.81	6.2673	1.3568	**<0.0001**
Lactose intolerant (*n* = 19)	SBTT (h)	3.28 ± 1.31	4.05 ± 1.67	3.81 ± 1.16	4.33 ± 1.79	−0.1262	0.495	0.8018
	CTT (h)	31.01 ± 5.92	27.10 ± 3.74	29.06 ± 6.17	32.64 ± 5.85	3.7462	1.3089	**0.0108**
	WGTT (h)	35.40 ± 5.61	32.72 ± 3.16	33.84 ± 6.04	37.85 ± 6.03	3.3441	1.3849	**0.0273**
Lactose tolerant (*n* = 21)	SBTT (h)	3.81 ± 1.55	4.00 ± 1.37	4.07 ± 2.82	3.27 ± 1.93	−0.4985	0.5583	0.3831
	CTT (h)	37.92 ± 9.17	28.87 ± 6.33	30.66 ± 9.95	40.60 ± 6.23	9.494	2.1656	**0.0003**
	WGTT (h)	42.55 ± 8.85	33.80 ± 6.80	35.48 ± 8.89	44.57 ± 6.36	8.9262	2.2947	**0.0010**

^a^Sequence 1: A1/A2 → A2

^b^Sequence 2: A2 → A1/A2

^c^Least squares mean difference (A1/A2 − A2)

^d^Values in bold are statistically significant at *P* < 0.05

*SD* standard deviation, *SBTT* small bowel transit time, *CTT* colonic transit time, *WGTT* whole gastrointestinal transit time

When data for each sequence were pooled according to the type of milk consumed, the consumption of milk containing both β-casein types was associated with significant increases in gastrointestinal transit times (see Additional file 2) as well as significant increases in serum IL-4, IgE and log IgG1 and decreases in fecal SCFAs (see Additional file 3).

### Adverse events

Thirteen episodes of diarrhea were reported in 10 (22.2 %) of 45 subjects. Eight events occurring in five subjects were considered related to consumption of milk containing both β-casein types, three events in three subjects were considered related to milk containing the A2 β-casein type, and two events in two subjects were considered unrelated to the interventions. Other adverse events included cough and cold in three and two subjects, respectively, but these were not considered related to the interventions.

## Discussion

For this study, we hypothesized that consumption of milk containing A1 β-casein would result in an increase in systemic inflammation (as characterized by serum biomarkers of inflammation) and be associated with gastrointestinal disorders similar to those of lactose intolerance in a cohort of subjects with perceived or confirmed lactose intolerance. We also hypothesized that elimination of the A1 β-casein type by providing subjects with milk that only contained the A2 β-casein type would avoid these effects of A1 β-casein. Consistent with our hypothesis, this crossover study revealed that consumption of the milk containing A1 β-casein was associated with greater gastrointestinal symptoms, higher concentrations of inflammation-related biomarkers and lower total fecal short-chain fatty acids, longer transit time, and longer responses and increased error rates on the SCIT compared with milk containing only the A2 β-casein type. Compositionally, the two products were nearly identical, except for the β-casein content where one contained only the A2 β-casein type while the ratio of the A1 and A2 β-casein types was 40:60 in milk containing both types (approximately 400 and 600 mg per 100 mL of milk). 

The observation that milk containing both types of β-casein increased serum IL-4 and other inflammatory markers are consistent with those reported by Ul Haq et al. [5]. They reported that A1-like types of β-casein (A1/A1 and A1/A2) induced inflammatory responses in the gastrointestinal tract of mice by activating the Th2 pathway, as illustrated by increases in myeloperoxidase, monocyte chemoattractant protein-1, antibodies (IgE, IgG, IgG1, and IgG2a), and Toll-like receptors 1 and 2, and increased leukocyte infiltration into the intestine [5]. They also reported that these effects were driven by BCM-7 and BCM-5 [26]. This cytokine/immune response reported by Ul Haq et al. and observed in the present study is also consistent with the intolerance-type reactions associated with asthma and eczema [27].

Consumption of milk containing both β-casein types was also associated with significantly lower SCFA concentrations than the consumption of milk containing only the A2 β-casein type. These results suggest that A1 β-casein consumption leads to reduced SCFA levels. SCFAs are fermentation products of gut biota [28] that have anti-inflammatory effects [29, 30] and enhance colonic cell function [31]. Accordingly, the consumption of A2 β-casein at the exclusion of A1 β-casein is expected to support microbial SCFA production, and hence avoid impairments in colonic health attributed to low SCFA production.

Gastrointestinal symptoms associated with the consumption of milk containing only the A2 β-casein type and milk containing only the A1 β-casein type (750 mL/day) were also evaluated in an 8-week crossover study conducted by Ho et al. [20]. In that study, consumption of milk containing the A1 β-casein type was associated with significantly higher Bristol Stool Scale scores compared with consumption of milk containing the A2 β-casein type. Moreover, the abdominal pain score was significantly correlated with stool consistency when subjects consumed milk containing the A1 β-casein type (*r* = 0.520, *P* = 0.001) but not milk containing the A2 β-casein type (*r* = −0.13, *P* = 0.43). Similarly, we observed greater gastrointestinal symptom scores together with longer gastrointestinal transit times, softer stools, and diarrhea when the subjects consumed milk containing both β-casein types as compared with milk containing only the A2 β-casein type. The results of both studies provide evidence that consumption of milk containing the A1 β-casein type may adversely affect gastrointestinal function and that exclusion of this type may alleviate these symptoms.

The present study also revealed that consumption of milk containing both β-casein types was associated with longer gastrointestinal transit times, particularly CTT and WGTT than the consumption of milk containing only the A2 β-casein type. These results are consistent with those reported by Barnett et al. in a rodent model [4]. They used titanium dioxide as a marker for gastrointestinal transit time. By contrast, a smart pill was used in the current study, and allowed us to obtain more accurate estimates of the total gastrointestinal transit time, as well as the transit times through specific regions of the intestine [32].

The present study revealed that the consumption of milk containing only the A2 β-casein type was not associated with worsening of any of the evaluated variables, and the results obtained after 2 weeks of consumption were comparable with those at baseline (i.e., after a 2-week washout of dairy products). In other words, the A1 β-casein type, but not the A2 β-casein type, had a negative impact on gastrointestinal function.

Using the smart pill, we also observed an increase in small bowel inflammation when the subjects consumed milk containing both β-casein types as compared with the consumption of milk containing only the A2 β-casein type. These results are consistent with the changes in inflammation-related biomarkers. However, changes were not apparent in all of the subjects and the P-value was of borderline significance (*P* = 0.042). It is possible that the intervention period was too short to elicit inflammation in many subjects. Therefore, these findings warrant further examination in a larger cohort or with a longer intervention time.

Finally, using the SCIT, we found that consumption of the milk containing both β-casein types was associated with small but highly significant increases in response time and error. The increases in response time were primarily found for the longer stimulus durations (the tail) while increases in error rate were largely restricted to the shorter stimulus durations (the head). This suggests that the consumption of milk containing both β-casein types is associated with reduced efficiency of preconscious automatic processing, but the longer stimulus duration-controlled processes help to reduce the deficit in processing efficiency at the cost of processing speed. This minor impairment of cognitive function can have a considerable impact in situations where rapid stimulus detection and/or rapid decision-making are required. This finding demonstrates that consumption of milk containing the A1 β-casein type affects more than just the gastrointestinal system; there are also effects on neural function. This finding is similar to the cognitive impairment observed in patients with undiagnosed celiac disease [33], and its explanation may lie with the increases in serum inflammation-related markers associated with the consumption of milk containing both β-casein types relative to milk containing only the A2 β-casein type. Elevated levels of circulating inflammatory markers have been linked to significant impairments in memory, attention, executive function and processing speed, even after controlling for age and other health-related factors [34–37].

Many of the symptoms associated with the consumption of A1 β-casein type are also associated with lactose intolerance [38]. It is notable that lactose intolerance or lactose malabsorption is comorbid to intestinal inflammation. Therefore, it is feasible that some people with perceived lactose intolerance may actually show adverse responses to the A1 β-casein type and peptides formed by its proteolysis [39, 40].

In the present study, the subjects underwent urinary galactose tests and about half of the subjects yielded positive results for lactose intolerance. Therefore, we compared the effects of both milk products on the severity of gastrointestinal symptoms and gastrointestinal transit times between subjects with lactose intolerance and subjects with lactose tolerance. Intriguingly, we found that the consumption of milk containing both β-casein types was associated with significant increases in both symptom severity and gastrointestinal transit times in both groups of subjects compared with baseline values. Notably, the observed changes were numerically greater in lactose intolerant subjects than in lactose tolerant subjects. By contrast, the consumption of milk containing only the A2 β-casein type did not increase gastrointestinal symptoms compared with the baseline values obtained after a 2-week washout period. Meanwhile, the increases in gastrointestinal transit times associated with the consumption of milk containing both β-casein types were slightly smaller in lactose intolerant subjects than in lactose tolerant subjects. However, the consumption of milk containing the A2 β-casein type was not associated with marked differences in gastrointestinal transit time between the lactose intolerant and lactose tolerant subjects. These findings suggest that some of the adverse gastrointestinal effects of dairy products may be due to the consumption of dairy containing A1 β-casein [39, 40]. This is because the consumption of milk containing A2 β-casein did not worsen these symptoms in lactose intolerant subjects relative to the baseline values after a washout or compared with the symptoms in lactose tolerant subjects. Both milk products contained equal amounts of lactose (4.8 %), which reinforces the concept that the differences in outcomes were driven by the presence or absence of A1 β-casein.

Several studies have revealed associations between A1 β-casein/BCM-7 and neurological problems, such as autism [41–44] and schizophrenia [45–49]. It has also been reported that elevated BCM-7 immunoreactivity is associated with delayed psychomotor development in infants [50]. The present data imply that A1 β-casein and its peptide derivatives also affect information processing in the brain. It has also been demonstrated that food-derived opioid peptides have a variety of direct effects on neural cells, including the expression of genes involved in redox and methylation processes, and epigenetic regulation [19]. It was postulated that milk-derived peptides may induce inflammation and systemic oxidation, including in the central nervous system [19], and these effects might impact on development or information processing. Further studies are necessary to confirm these effects and elucidate the underlying pathway.

Some limitations warrant mention. First, the smart pill was not used at baseline, so it is not possible to determine whether milk containing only the A2 β-casein type influenced gastrointestinal transit time beyond the effects of washing out of dairy products. Second, the duration of each intervention period (2 weeks) may have been too short to elicit changes in some biomarkers or local inflammation. Therefore, longer interventions may be necessary to provide more reliable estimates of the effects of milk containing both β-casein types, as well as the beneficial effects of milk containing the A2 β-casein type on gastrointestinal function. Third, we used milk containing both the A1 and A2 β-casein types (40:60), because milk containing only the A1 β-casein type was unavailable. However, as the A2 β-casein type is thought to be “inert” in terms of opiate receptor agonism [4], the presence of A2 β-casein is unlikely to confound the effects or may alleviate the potentially deleterious effects of the A1 β-casein type. Finally, this study focused solely on gastrointestinal symptoms, so any additional effects of the investigated products could not be tested.

## Conclusions

In conclusion, this study has demonstrated that consumption of milk containing A1 β-casein in addition to A2 β-casein worsens gastrointestinal symptoms, increases gastrointestinal transit time, increases serum inflammation markers, lowers total fecal SCFA content, slows cognitive processing speed and decreases processing accuracy compared with the baseline values. Consumption of milk containing only A2 β-casein did not adversely affect these variables, indicating that the changes observed with milk containing both β-casein types were attributable to the presence of A1 β-casein. Furthermore, consumption of milk containing both types was associated with greater worsening of gastrointestinal symptoms and gastrointestinal transit time in lactose intolerant subjects than in lactose tolerant subjects, whereas milk containing only A2 β-casein did not exacerbate these symptoms in lactose intolerant subjects. These results suggest that the exacerbation of gastrointestinal symptoms associated with milk in lactose intolerant subjects may be related to A1 β-casein rather than lactose per se.

## Additional files

Additional file 1: Exclusion criteria, Laboratory tests, Adverse event codes. (PDF 1.39 mb)

Additional file 2: Regional gastrointestinal transit times according to the product type in lactose intolerant and lactose intolerant subjects. (PDF 469 kb)

Additional file 3: Blood and fecal laboratory tests according to the product type in lactose intolerant and lactose intolerant subjects. (PDF 507 kb)

## Abbreviations

BCM: β-casomorphin
CTT: colonic transit time
ECG: electrocardiogram
PD3: post-dairy digestive discomfort
SCFA: short-chain fatty acid
SCIT: Subtle Cognitive Impairment Test
SBTT: small bowel transit time
WGTT: whole gastrointestinal transit time
