# Erratum to: ‘Effects of milk containing only A2 beta casein versus milk containing both A1 and A2 beta casein proteins on gastrointestinal physiology, symptoms of discomfort, and cognitive behavior of people with self-reported intolerance to traditional cows’ milk’

**DOI:** 10.1186/s12937-016-0164-y

**Published:** 2016-04-29

**Authors:** Sun Jianqin, Xu Leiming, Xia Lu, Gregory W. Yelland, Jiayi Ni, Andrew Clarke

**Affiliations:** 1 Clinical Nutrition Center, Huadong Hospital affiliated to Fudan University, Shanghai, China; 2 Department of Gastroenterology, Xin Hua Hospital affiliated to Shanghai Jiao Tong University School of Medicine, Shanghai, China; 3 Endoscopic Center, Shanghai Internal Medicine Center, Shanghai, China; 4 Department of Gastroenterology, Central Clinical School, The Alfred Centre, Monash University, Melbourne, VIC Australia; 5 School of Health Sciences, RMIT University, Bundoora, VIC Australia; 6 S.P.R.I.M. China (Shanghai) Consulting Co., Ltd, Shanghai, China; 7 The a2 Milk Company Limited, Auckland, New Zealand

Unfortunately, the original version of this article [1] contained an error. Figure 1 has been updated since it was last uploaded. BCM-7, GSH and MPO have all been removed and PD3 is replaced by VAS. The figure is included here. The figure legend has also been changed. It used to read as:

**Fig. 1:**
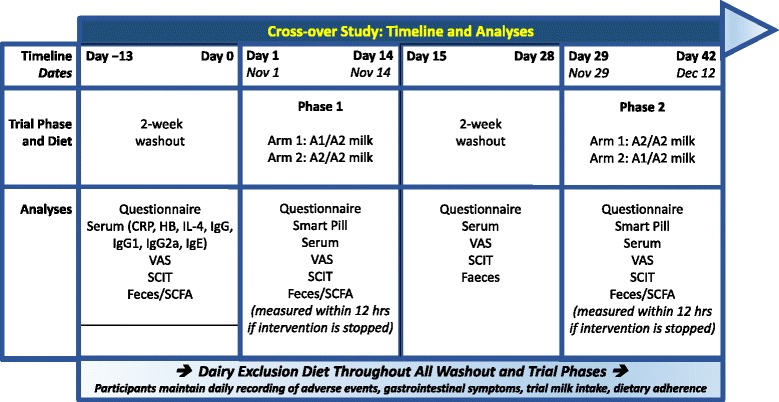
Study design. A1 = milk containing A1 and A2 β-casein; A2 = milk containing only A2 β-casein; hs-CRP, highly sensitive C-reactive protein; Hb, hemoglobin; IL-4, interleukin-4; Ig, immunoglobulin; VAS, visual analog scale scores; SCIT, Subtle Cognitive Impairment Test; SCFA, short-chain fatty acids

Study design. A1 = milk containing A1 and A2 β-casein; A2 = milk containing only A2 β-casein; hs-CRP, highly sensitive C-reactive protein; Hb, hemoglobin; IL-4, interleukin-4; Ig, immunoglobulin; BCM-7, β-casomorphin-7; GSH, glutathione; PD3, gastrointestinal symptoms of post-dairy digestive discomfort; SCIT, Subtle Cognitive Impairment Test; SCFA, short-chain fatty acids; MPO, myeloperoxidase.

It has now been changed to read as follows:

Study design. A1 = milk containing A1 and A2 β-casein; A2 = milk containing only A2 β-casein; hs-CRP, highly sensitive C-reactive protein; Hb, hemoglobin; IL-4, interleukin-4; Ig, immunoglobulin; VAS, visual analog scale scores; SCIT, Subtle Cognitive Impairment Test; SCFA, short-chain fatty acids.

There will be an update for these corrections as well as this erratum.
