# Supplementary material for: Effects of milk containing only A2 beta casein versus milk containing both A1 and A2 beta casein proteins on gastrointestinal physiology, symptoms of discomfort, and cognitive behavior of people with self-reported intolerance to traditional cows’ milk
Source: Nutr J. 2016 Apr 2;15:35. doi: 10.1186/s12937-016-0147-z (PMC4818854; doi:10.1186/s12937-016-0147-z)
Supplement: Supplementary file 1 — Exclusion criteria, Laboratory tests, Adverse event codes. (PDF 1.39 mb) [file 12937_2016_147_MOESM1_ESM.pdf]

Additional Material

Exclusion criteria

The exclusion criteria were as follows: pregnant or breastfeeding women; known dairy allergy; severe intolerance to milk; history of fecal impaction; trying to lose weight by following a diet or exercise regimen designed for weight loss, or taking any drug influencing appetite and any drug for weight loss within 3 months before screening; participation in similar interventions of dairy or probiotic-containing product within 3 months before screening; currently taking drugs for the treatment of cardiovascular or metabolic diseases; history of gastrointestinal disorders, liver disease, nephropathy, endocrine disease, blood disorders, respiratory and cardiovascular diseases likely to influence the study results; current or history of alcohol abuse, or use of illicit drugs, substances or over the counter/prescription drugs likely to affect gastrointestinal function; currently suffering from any gastrointestinal disorders or gastrointestinal disease, such as irritable bowel syndrome, colitis, ulcerative colitis, and celiac disease; hospitalization for any reason within 3 months before screening; and according to the investigator's judgment, current use of drugs likely to affect gastrointestinal function or immune system.

Laboratory tests

Urinary galactose test

For the urinary galactose test, after an overnight fast and emptying their bladder, the subjects consumed 485 ml of milk. Urine samples were collected at 1 and 2 h after milk consumption. Samples were purified within 30 min of collection and the presence of galactose was determined using a colorimetric assay.

Measurement of serum biomarkers

Blood samples were obtained at 7.30–8.00 am after an overnight fast to measure serum biomarkers using enzyme-linked immunosorbent assays (Cusabio Biotech Co., Ltd., Wuhan, China) for interleukin-4 (IL-4), immunoglobulin (Ig) G1, and IgG2a; immunoturbidemetry for IgG and high-sensitivity C-reactive protein (hs-CRP; Roche P800 Biochemical Immune Analyzer; Roche Diagnostics, Basel, Switzerland); electrochemiluminesence assays for IgE (Roche Diagnostics); and a cyanide-free sodium lauryl sulfate photometry for hemoglobin (SYSMEX XS-1000I; Sysmex Corp., Kobe, Japan).

Measurement of fecal biomarkers

Fecal samples were collected to measure the short chain fatty acid (SCFA) content using an Agilent 6890N (Agilent Technologies, Inc., Santa Clara, CA, USA).

Adverse event codes

Whole body

BODY 010 Anemia

BODY 002 Dehydration

BODY 005 Infection not classified elsewhere

BODY 001 Irritability/fussiness/excessive crying

BODY 019 Reaction to vaccine

BODY 008 Rule out sepsis

BODY 009 Sepsis

Cardiovascular

CARD 004 Heart murmur

Eyes, ears, nose, and throat

EENT 037 Allergic rhinitis

EENT 038 Allergic rhino-conjunctivitis

EENT 006 Conjunctivitis

EENT 018 Eye movement disorder

EENT 013 Hearing deficit

EENT 015 Infectious rhinitis/sinusitis

EENT 002 Nasal congestion/nasopharyngeal congestion with no other related symptoms

EENT 030 Otitis externa

EENT 001 Otitis media

EENT 044 Serous otitis media

Gastrointestinal

GI 008 Acute gastroenteritis / diarrheal disease

GI 043 Allergic colitis

GI 044 Allergic gastroenteritis

GI 010 Anal fissure

GI 009 Bloody stool

GI 007 Colic

GI 006 Constipation

GI 020 Cows' milk intolerance

GI 005 Diarrhea

GI 001 Emesis/vomiting

GI 017 Frequent stools

GI 004 Gas

GI 003 G.E. reflux

GI 016 Pyloric stenosis

GI 034 Rectal stenosis

GI 019 Umbilical hernia

Metabolic and nutrition

MAN 008 Failure to thrive

MAN 002 Lack of appetite

MAN 007 Poor weight gain/weight loss

Musculoskeletal

MS 004 Deformity

MS 001 Fracture

MS 005 Torticollis

MS 003 Trauma

Nervous system

NER 002 Meningitis

NER 001 Seizure/convulsion

Respiratory

RESP 009 Bronchiolitis

RESP 005 Bronchitis

RESP 002 Cough with no other related symptoms

RESP 004 Croup/laryngitis

RESP 012 Pharyngitis

RESP 007 Pneumonia

RESP 011 Respiratory syncytial virus

RESP 024 Tonsillitis

RESP 001 Upper respiratory infection/cold/respiratory viral infection

RESP 006 Wheezing/reactive airway disease

Skin

SK 033 Abscess

SK 012 Chickenpox

SK 001 Diaper rash

SK 003 Dry skin

SK 004 Eczema / atopic dermatitis

SK 028 Fungal skin infection

SK 006 Impetigo

SK 010 Other rash

SK 005 Seborrhea/cradle cap

SK 009 Urticaria

SK 002 Yeast infection

Urogenital

UG 017 Hydrocele

UG 005 Inguinal hernia

UG 013 Penile adhesion

UG 004 Urinary tract infection

UG 001 Vaginal discharge

Other

OTH 001 Other
