# Supplementary material for: Effects of milk containing only A2 beta casein versus milk containing both A1 and A2 beta casein proteins on gastrointestinal physiology, symptoms of discomfort, and cognitive behavior of people with self-reported intolerance to traditional cows’ milk
Source: Nutr J. 2016 Apr 2;15:35. doi: 10.1186/s12937-016-0147-z (PMC4818854; doi:10.1186/s12937-016-0147-z)
Supplement: Supplementary file 2 — Regional gastrointestinal transit times according to the product type in lactose intolerant and lactose intolerant subjects. (PDF 469 kb) [file 12937_2016_147_MOESM2_ESM.pdf]

Additional Material

Additional Table Regional gastrointestinal transit times according to the product type in lactose intolerant and lactose intolerant subjects

	**Variable**	**A1/A2**[a]	**A2**[b]	**1. Estimate**[c]	**2. SD**	**3. *P*-value**[d]
Overall (n=40)	SBTT (h)	3.75 (1.64)	3.97 (1.65)	−0.2198	0.3652	0.5507
	CTT (h)	35.56 (7.89)	28.88 (6.49)	6.6737	1.2717	**<0.0001**
	WGTT (h)	40.18 (7.68)	33.89 (6.28)	6.2949	1.3333	**<0.0001**
**Lactose intolerant (n=19)**	SBTT (h)	3.89 (1.65)	3.91 (1.36)	−0.0249	0.4912	0.9602
	CTT (h)	31.96 (5.77)	28.24 (5.26)	3.7202	1.2567	**0.0084**
	WGTT (h)	36.82 (5.83)	33.37 (4.95)	3.4498	1.3381	**0.0189**
**Lactose tolerant (n=21)**	SBTT (h)	3.63 (1.66)	4.02 (1.90)	−0.3962	0.5171	0.4525
	CTT (h)	38.81 (8.25)	29.47 (7.52)	9.3458	1.9922	**0.0001**
	WGTT (h)	43.23 (8.00)	34.36 (7.38)	8.869	2.109	**0.0004**

[a]Milk containing both A1 and A2 β-casein type.

[b]Milk containing only A2 β-casein type.

[c]Least squares mean difference (A1/A2 − A2).

[d]Values in bold are statistically significant at $P<0.05$.

SD, standard deviation; SBTT,small bowel transit time; CTT, colonic transit time; WGTT, whole gastrointestinal transit time.
