# Supplementary material for: Effects of milk containing only A2 beta casein versus milk containing both A1 and A2 beta casein proteins on gastrointestinal physiology, symptoms of discomfort, and cognitive behavior of people with self-reported intolerance to traditional cows’ milk
Source: Nutr J. 2016 Apr 2;15:35. doi: 10.1186/s12937-016-0147-z (PMC4818854; doi:10.1186/s12937-016-0147-z)
Supplement: Supplementary file 3 — Blood and fecal laboratory tests according to the product type in lactose intolerant and lactose intolerant subjects. (PDF 507 kb) [file 12937_2016_147_MOESM3_ESM.pdf]

Additional Material

Additional Table Blood and fecal laboratory tests according to the product type in lactose intolerant and lactose intolerant subjects

Variable	A1/A2[a]		A2[b]		Estimate[c]	SE	*P*-value[d]
	BL	PI	BL	PI			
**Serum**							
hs-CRP (mg/L)	1.02 ± 0.85	1.18 ± 0.87	1.01 ± 0.82	1.08 ± 0.89	0.0728[e]	0.0373	0.0576
Hb (g/L)	139.2 ± 21.8	143.4 ± 17.7	139.6 ± 21.8	144.5 ± 17.0	−0.8662	1.654	0.6032
IL-4 (ng/L)	11.9 ± 3.7	14.3 ± 4.7	11.7 ± 3.8	11.6 ± 3.4	2.524	0.528	<0.0001
IgG (g/L)	10.5 ± 2.0	11.9 ± 2.0	10.4 ± 1.9	10.8 ± 1.7	0.1426[e]	0.039	0.0007
IgE (IU/mL)	58.0 ± 29.7	66.3 ± 35.8	59.9 ± 30.0	62.2 ± 30.4	5.9775	2.5446	0.0235
IgG1 (μg/mL)	31.7 ± 29.1	38.1 ± 34.8	32.6 ± 30.4	31.5 ± 30.4	0.2417[f]	0.0792	0.0039
BCM-7 (ng/mL)[g]	0.67	0.97	0.63	0.74			0.0171[h]
**Feces**							
Acetic acid (%)	0.41 (0.16)	0.39 (0.13)	0.40 (0.17)	0.45 (0.15)	−0.0673	0.0226	0.0048
Propanoic acid (%)	0.18 (0.08)	0.17 (0.07)	0.17 (0.08)	0.18 (0.11)	−0.0115[e]	0.0167	0.493
Butanoic acid (%)	0.17 (0.08)	0.16 (0.06)	0.16 (0.08)	0.21 (0.08)	−0.0518	0.0122	0.0001
Total SCFA (%)	0.75 (0.26)	0.72 (0.21)	0.74 (0.29)	0.85 (0.26)	−0.1301	0.0363	0.0009

[a]Milk containing both A1 and A2 β-casein type.

[b]Milk containing only A2 β-casein type.

[c]Least squares mean difference (A1/A2 − A2).

[d]Values in bold are statistically significant at $P<0.05$.

[e]Because the variable was non-normally distributed, mixed-effects ANOVA was performed using the square root-transformed values.

[f]Because the variable was non-normally distributed, mixed-effects ANOVA was performed using the log-transformed values.

[g]Median values.

[h]Wilcoxon signed rank test.

ANOVA, analysis of variance; BL, baseline; PI, postintervention (i.e., after 2 weeks of each intervention); SE, standard error; hs-CRP, highly sensitive C-reactive protein; Hb, hemoglobin; BCM-7, β-casomorphin-7; IL-4, interleukin-4; Ig, immunoglobulin; SCFA, short-chain fatty acids.
